# Entrainment of Lymphatic Contraction to Oscillatory Flow

**DOI:** 10.1038/s41598-019-42142-9

**Published:** 2019-04-09

**Authors:** Anish Mukherjee, Joshua Hooks, Zhanna Nepiyushchikh, J. Brandon Dixon

**Affiliations:** 1 School of Electrical and Computer Engineering, Georgia Institute of Technology, Atlanta, GA 30332 Georgia; 2 George W. Woodruff School of Mechanical Engineering, Georgia Institute of Technology, Atlanta, GA 30332 Georgia; 3 Coulter Department of Biomedical Engineering, Georgia Institute of Technology, Atlanta, GA 30332 Georgia

## Abstract

Lymphedema, a disfiguring condition characterized by an asymmetrical swelling of the limbs, is suspected to be caused by dysfunctions in the lymphatic system. A possible source of lymphatic dysfunction is the reduced mechanosensitivity of lymphangions, the spontaneously contracting units of the lymphatic system. In this study, the entrainment of lymphangions to an oscillatory wall shear stress (OWSS) is characterized in rat thoracic ducts in relation to their shear sensitivity. The critical shear stress above which the thoracic ducts show a substantial inhibition of contraction was found to be significantly negatively correlated to the diameter of the lymphangion. The entrainment of the lymphangion to an applied OWSS was found to be significantly dependent on the difference between the applied frequency and the intrinsic frequency of contraction of the lymphangion. The strength of the entrainment was also positively correlated to the applied shear stress when the applied shear was less than the critical shear stress of the vessel. The ejection fraction and fractional pump flow were also affected by the difference between the frequency of the applied OWSS and the vessel's intrinsic contraction frequency. The results suggest an adaptation of the lymphangion contractility to the existing oscillatory shear stress as a function of its intrinsic contractility and shear sensitivity. These adaptations might be crucial to ensure synchronized contraction of lymphangions through mechanosensitive means and might help explain the lymphatic dysfunctions that result from impaired mechanosensitivity.

## Introduction

The lymphatic system plays a crucial role in the regulation of tissue fluid balance and hence, the maintenance of interstitial fluid volume. The interstitial fluid that accumulates as a result of extravasation of fluid from the blood capillaries is cleared by the lymphatic system. Through a network of capillaries and collecting vessels, the lymphatic system takes up the excess interstitial fluid and transports it back into the blood circulation. The lymphatic system plays an important role in not only fluid homeostasis but also in lipid transport and the immune system. Dysfunctions of the lymphatic system are closely related to a condition called “lymphedema”, which is characterized by a persistent swelling of the tissue space (often the limbs) due to an accumulation of fluid^1–3^. Lymphedema has largely been understudied since it is not a life-threatening condition, but it has a severe impact on the quality of life of the patient. In addition to a gross swelling of the arms and/or legs, which is aesthetically unpleasant and can lead to psychological scarring for the patient, the freedom of movement of the limbs is restricted, leading to discomfort and hindered functionality. In recent years, the severity of lymphedema has been recognized by the research community and focus has been put on studying the underlying mechanisms governing this condition.

The onset and progression of lymphedema are coincident with a change in the contractile function of the lymphatic system, possibly due to aberrant mechanical forces^1–8^. Lymphangions, the basic functional units of the collecting lymphatic system exhibit intrinsic contractility. The responsiveness of lymphangions to mechanical stimuli has been well documented^9–19^. There are primarily two types of mechanical forces that need to be considered in the lymphatic system – the hoop stress exerted on the wall due to the transmural pressure and the wall shear stress (WSS) in the lumen of the collecting lymphatic vessels due to lymph flow. The effect of luminal pressure on the lymphangion is that of exerting a hoop stress (stretch) on the lymphatic endothelial and muscle cells, and its impact on the physiology of the collecting vessels has been studied widely^15,17,20–22^. The transmural pressure has a direct influence on the contraction frequency, the end systolic and diastolic volume of the vessel, as well as other pumping metrics like ejection fraction. The general trend is an increase in the contraction frequency and pump function in response to an elevated transmural pressure from zero to some optimal pressure, after which a further increase in pressure causes a reduction in pump function^6,17,21,23–27^.

Shear stress is an important factor in modulating both the tonic and phasic contraction of the lymphangions^11,13,16,19,21^, but has been less widely studied compared to the effect of transmural pressure on lymphatic pump function. WSS is directly related to the lymph flow rate through the vessel and is also dictated by the vessel diameter. An elevation in lymph flow rate and reduction in the vessel diameter corresponds to an increased WSS. Experimentally, it is easier to control the pressure gradient across the vessel than the flow rate through it and hence studies have mostly focused on using the pressure gradient as a means to impose WSS on the vessel. Gashev *et al*. showed that both the chronotropic and inotropic response of the lymphatic vessels are negatively affected by an imposed pressure gradient^19^. Numerous studies since these original ones have confirmed the findings of Gashev *et al*., that imposed flow decreases both the contraction amplitude and the frequency^13,28–32^. It was found that with an increase in the applied pressure gradient in rat mesenteric lymphatic vessels the contraction frequency decreases, but this decrease is only temporary since the frequency recovers over time with the same applied pressure gradient. The contraction amplitude decreases due to an increase in the end-systolic diameter. This effect is more pronounced in rat thoracic ducts than the mesenteric lymphatics^33^. These changes are also dependent on the rate of change of the flow, with a higher rate of change producing a larger change in the lymphatic pumping parameters^13,15^.

The region chosen for the study can affect the response of the lymphatic vessels to transmural pressure and WSS. For example, contraction frequency was found to be more heavily dependent on transmural pressure for rat mesenteric and cervical lymphatics as opposed to the thoracic duct^33^. Rat thoracic ducts also significantly inhibit their contraction with large, favorable pressure gradients, while rat mesenteric and cervical lymphatics still exhibit contraction at these pressure differences^19,34^. Since these studies controlled the flow through the vessel by controlling the pressure at the inlet and the outlet of the cannulae, they did not take into consideration the exact WSS being applied, or the fact that the relation between the applied pressure difference and the WSS is highly dependent on the diameter of the vessel and the pressure drop that occurs along the pipette tips used for cannulating the vessels. Thus it is important to investigate the mechanosensitivity of the lymphangions with respect to the WSS experienced by the lymphangion.

While the response of lymphatic vessels to oscillatory wall shear stress (OWSS) have been explored before^13^, we show in this study that the response is more nuanced than previously believed. A platform is introduced to investigate the response of lymphangions to an OWSS in the frequency domain using continuous wavelet transform. This platform allows for the analysis of the response of the lymphatic vessels to dynamic forces by looking at their frequency domain response, which allows for the quantification of their entrainment to externally applied oscillatory forces. The ability of a lymphatic vessel to entrain its contraction to an externally applied OWSS is hypothesized to be dependent on its sensitivity to the WSS and its intrinsic contraction frequency (the average frequency with which the vessel contracts when no flow is applied to its lumen). The specific parameters of the applied OWSS (frequency and amplitude) are also hypothesized to affect the overall pumping performance of the lymphatic vessels through the mechanism of entrainment that is elucidated in this work. The entrainment of the lymphangions was found to be dependent on the external shear stress as well as intrinsic contractility and mechanosensitivity of the lymphangions. Optimal external shear stress conditions were also found to exist that maximized lymphatic pumping efficiency.

## Results

### Characterization of wall shear stress sensitivity

**Figure 1:**
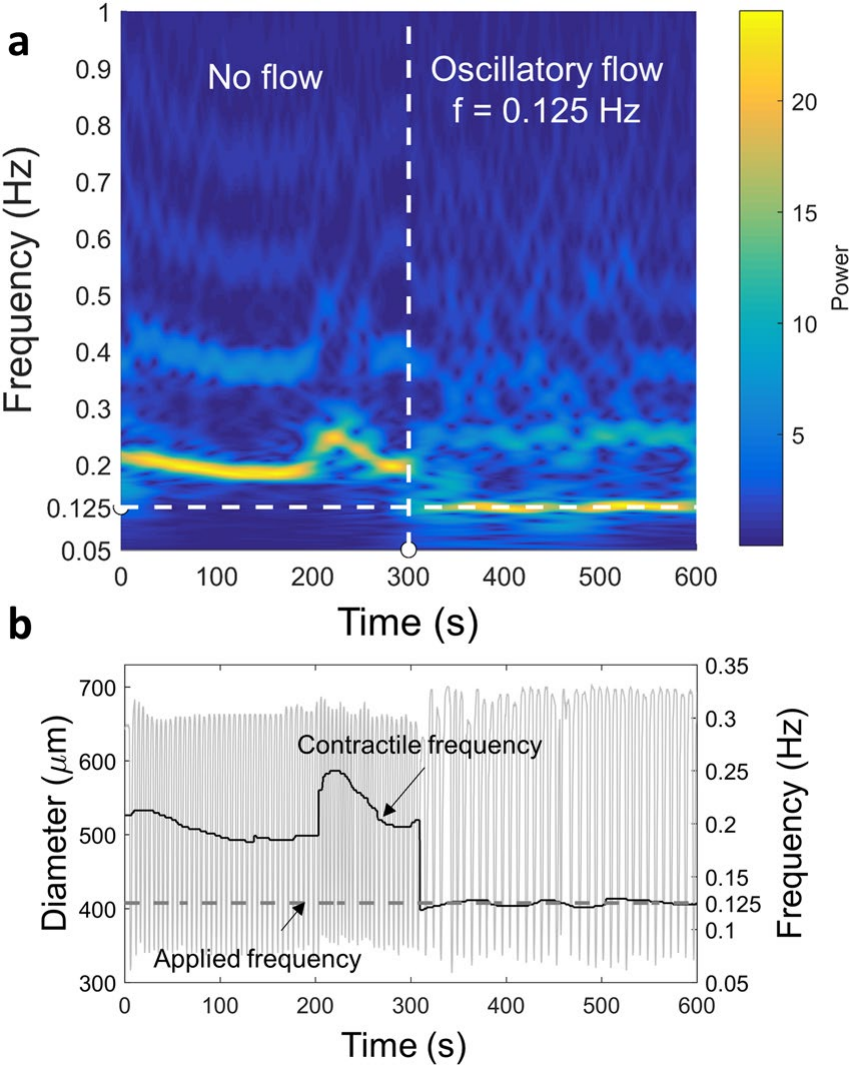
The method for analyzing the data in the frequency domain is shown. (**a**) Continuous Wavelet Transform (CWT) provides a detailed frequency domain information. The colorbar represents the magnitude of the CWT coefficient which is referred to as the power. The vertical dashed line represents the time at which imposed oscillatory flow begins and the horizontal dashed line represents the frequency of the imposed flow. (**b**) The frequency information can be easily isolated from the CWT. The calculated frequency is shown as a function of time and is overlaid over the diameter variations. For this particular experiment, the vessel was not exposed to flow at the start of the experiment and at 300 seconds an imposed oscillatory flow was applied to the vessel at the frequency indicated in the figure.

To determine the wall shear stress sensitivity, the isolated lymphatic vessels were exposed to favorable pressure gradients that were linearly increased in magnitude over fixed time intervals (0 to 9 cmH_2_O over 5 min and 15 min) while holding the transmural pressure constant at 3 cmH_2_O, while video of the contracting vessel was recorded through a microscope. Vessel contraction frequency over time was calculated using a continuous wavelet transform (CWT), which is a frequency domain analysis technique that provides the spectral content of a waveform as a function of frequency and time. The magnitude of the wavelet coefficients represents the presence of a particular frequency component in the waveform at a particular time. An example spectrogram is shown in Fig. 1a for a diameter tracing of a contracting vessel at a single location along the vessel. The colorbar represents the magnitudes of the CWT coefficients. At any particular point in time, the frequency at which the magnitude of the coefficient (hereby referred to as “power”) is the highest was defined as the “dominant frequency” at that time point, which is represented in Fig. 1b. The other peaks in the CWT represent other prominent frequency components in the diameter tracing, which can occur at the harmonics. The use of CWT allows for the subsequent analyses of the response of the lymphangion to different OWSS conditions, by studying the relative magnitudes of the different frequency components as a function of the frequency and amplitude of the applied OWSS.

From previous work it is known that the WSS that is necessary to inhibit contraction in thoracic duct segments is quite variable between thoracic duct segments from different rats^13^. Hence it is important to establish the wall shear stress sensitivity of each vessel prior to exposing them to OWSS in order to determine the extent to which each vessel’s unique shear sensitivity is responsible for entrainment to oscilliatory flow. To this end, the response of the thoracic duct to a ramped shear stress was analyzed with CWT to obtain the shear sensitivity information. The dominant frequency was obtained as a function of time from the CWT of the diameter tracings for the ramp experiment. The imposed WSS was then obtained as a function of time for the ramp protocol by reading the syringe position of the perfusion system, assuming Poisuielle flow and using the diastolic diameter of the vessel as described previously^13^. A linear fit to this data was obtained, assuming that the imposed WSS is zero at the start of the shear ramp.

**Figure 2:**
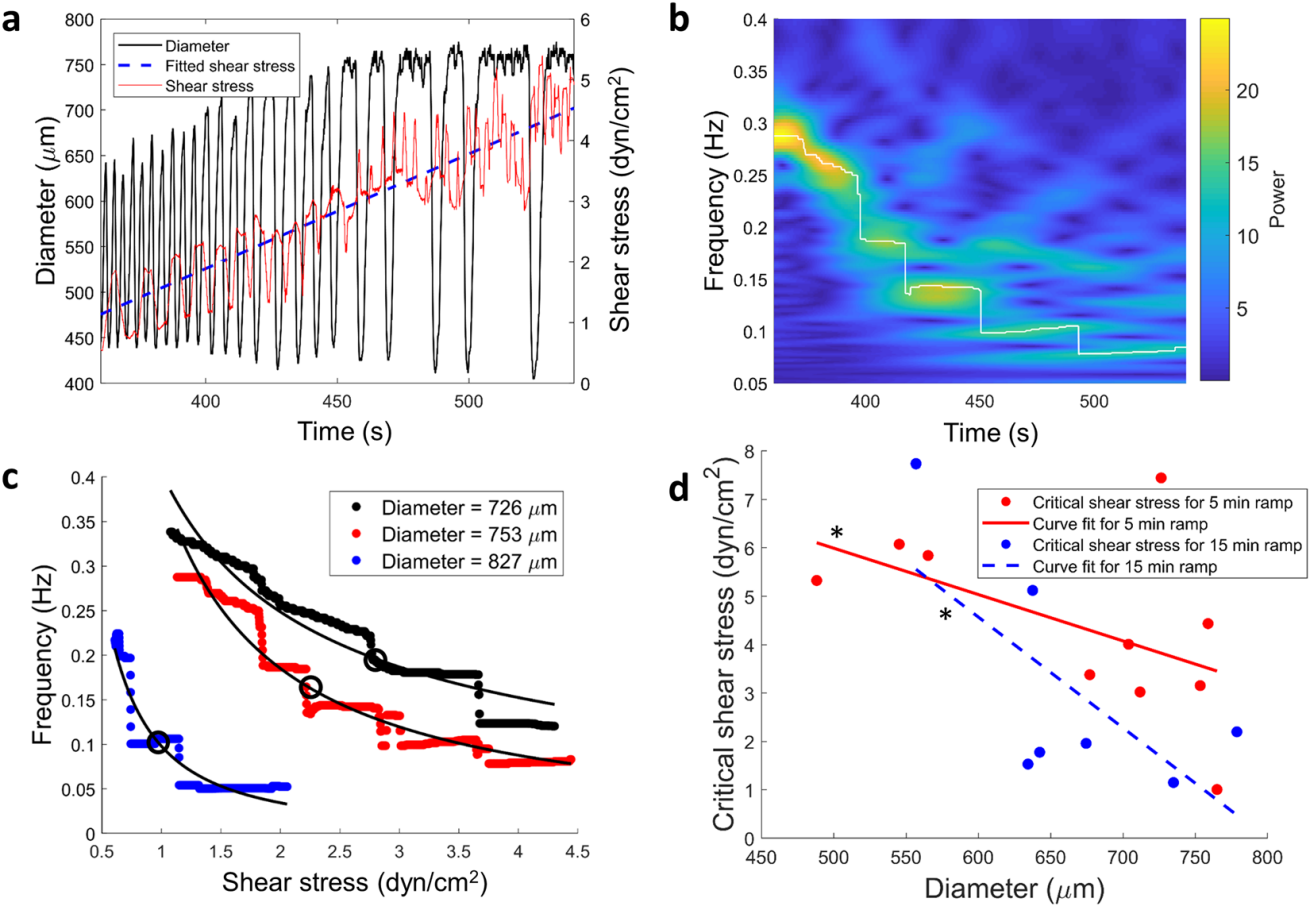
The critical wall shear stress of the vessel negatively correlates with the passive vessel diameter. (**a**) The diameter tracing is shown (in black) along with the applied WSS (in red) and fitted WSS (in blue). The contraction frequency of the vessel goes down with time when a ramped WSS is applied to the vessel. (**b**) The CWT spectrogram of the diameter tracing is provided, with the white tracing highlighting the dominant frequency of contraction. The decrease in frequency with shear stress can be quantified from the dominant frequency information. (**c**) The frequency is plotted as a function of the shear stress, and a power law curve is fitted to the data. The critical shear stress points, where the frequency is half of the intrinsic frequency are marked with the black circles. Representative fitted curves are shown for three different vessels and a dependence on the diameter of the vessel can be readily seen. (**d**) The critical shear stress is plotted as a function of the diameter of the thoracic duct for the two different shear stress ramps applied to the vessels (N = 9 for 5 min WSS ramp and N = 7 for 15 min WSS ramp) and the significance of the correlation is represented by the asterisk (*). Linear regression curves are fitted to the data and show a significant negative correlation (Spearman correlation coefficient of −0.7454 and −0.787 with p values of 0.0133 and 0.0357 respectively for 5 min and 15 min ramps). The regression does not depend significantly on the rate of the ramp applied (p = 0.8629).

With the fitted shear stress data being obtained, the dominant frequency was then plotted as a function of the shear stress to obtain the shear sensitivity (Fig. 2). The relationship between frequency and WSS appears to follow a power law function of the form f = at^b^, where f is the frequency, t is the time, and a and b are the parameters to be optimized. Curves of this nature fitted to the data had an average r-squared value of about 0.9, which indicated that the power law model provides a good approximation to the shear-frequency relationship over the ranges tested.

The shear sensitivity of the vessel was represented by a “critical shear stress”, which was defined as the shear stress at which the frequency of contraction drops down to half of the intrinsic frequency of contraction of the vessel. The critical shear stress was obtained from the power law relationship relating the frequency of contraction to the imposed WSS. The shear sensitivity is inversely related to the critical shear stress. The critical shear stress ranged between 0.1 to 10 dynes/cm^2^ and was found to be significantly correlated to the average diastolic diameter of the vessel, irrespective of the rate of the ramp waveform applied to the vessel. The Spearman correlation coefficient was calculated between the critical shear stress and the diastolic diameter and were found to be −0.7454 and −0.787, with p values of 0.0133 and 0.0357 respectively for the ramp waveforms applied for 5 min and 15 min. The significance of the correlation coefficient was calculated using Fisher’s r-to-z transformation. The shear sensitivity was not found to be significantly related to the rate of the ramped shear stress applied to the vessel (p = 0.8434).

### Sensitivity of entrainment to OWSS parameters

To study the effect of variations in the frequencies and amplitudes of the OWSS on the response of the lymphangion, the spectral distribution of power and how it changes with the frequency and amplitude of the shear stresses applied to the vessel was investigated using CWT. The percentage of power at a particular frequency is representative of the prevalence of that frequency component compared to the other components. The percentage of power at the applied frequency is thus indicative of the “strength of entrainment” of the vessel to the applied frequencies. Vessels were exposed to three different frequencies of imposed flow (0.075 Hz, 0.2 Hz, and 0.35 Hz). These values were chosen to be reflective of frequencies that were less than, comparable to, and above the typical intrinsic contraction frequency of isolated pressurized rat thoracic ducts. These three frequencies were also applied with three different amplitudes of the imposed pressure gradient waveform (${\rm{\Delta }}P$ = 4, 6, and 8 cm H_2_0, where ${\rm{\Delta }}P$ is the pressure difference measured between the inlet and the outlet pipettes on which the vessel is cannulated).

**Figure 3:**
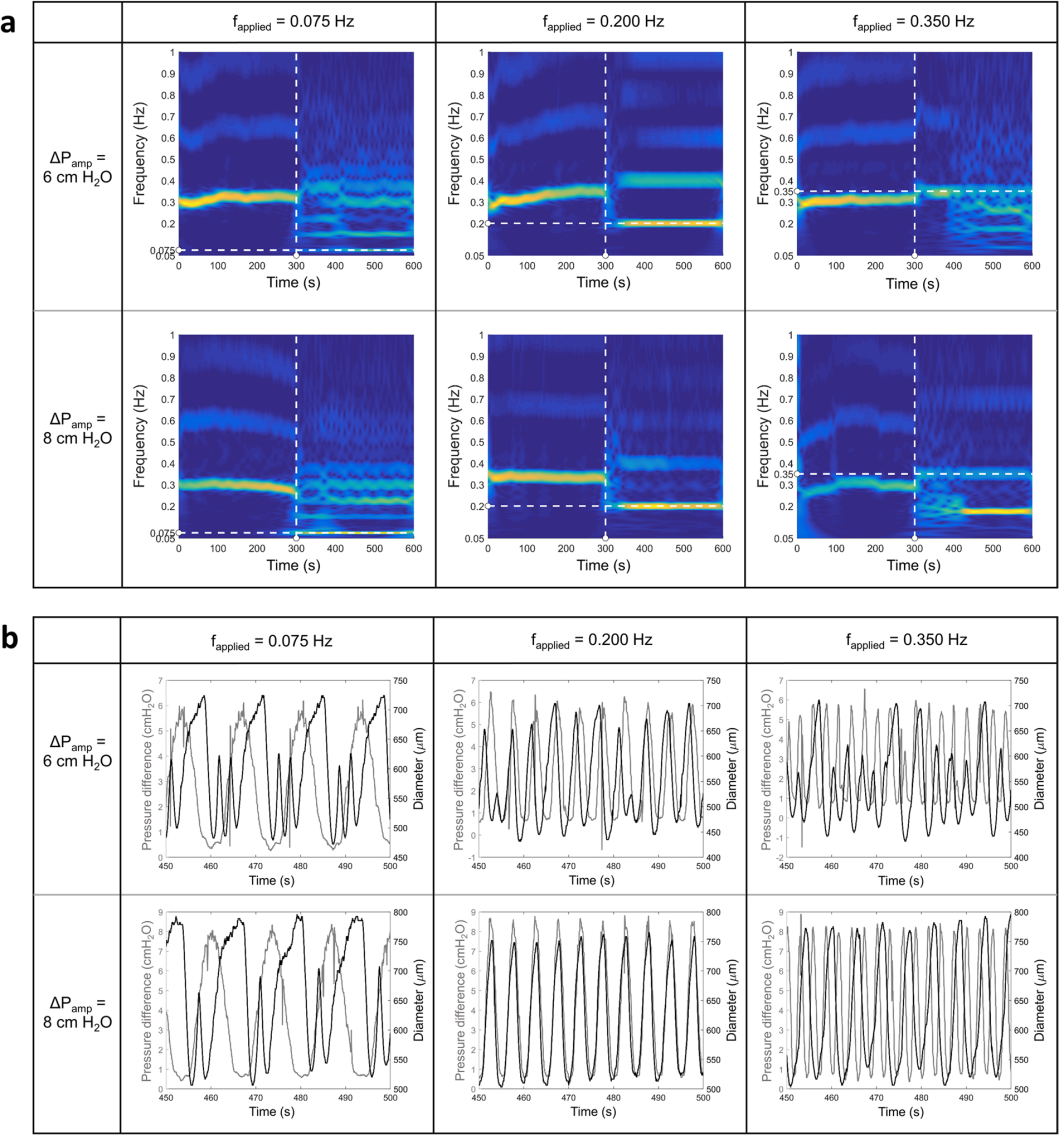
Representative CWT spectrograms, and diameter and pressure gradient tracings are shown for the three applied frequencies of 0.075 Hz, 0.2 Hz, and 0.35 Hz, and two pressure gradients having amplitudes of 6 cmH_2_O and 8 cmH_2_O. (**a**) The vertical dashed line represents the time at which the OWSS was applied and the horizontal dashed line represents the frequency of the applied OWSS. The graphs show a clear dependence of the entrainment, represented by the power in the applied frequency component, with the applied frequency (comparing between the columns) and the magnitude of the shear (comparing between the rows). When the applied frequency exceeds the intrinsic frequency of contraction of about 0.3 Hz (bottom right panel), the maximum power is seen to be localized at half the applied frequency instead of the applied frequency. (**b**) The pressure and diameter tracings for the vessel are overlaid, with pressure being represented by the gray line and diameter by the black line. The dependence of the entrainment on the applied frequency and shear stress is represented by the pressure and diameter tracings. When the applied frequency is greater than the intrinsic frequency of contraction, the vessel contracts at half the applied frequency (bottom right panel).

An observation of the CWT spectrograms for a single vessel at different applied frequencies and amplitudes, as shown in Fig. 3a, reveals that the extent of entrainment of the vessel depends on both the frequency of the applied waveform and the magnitude of the pressure gradient amplitude. An interesting thing to note is that for an applied frequency of 0.35 Hz, the maximum power did not occur at the applied frequency, but rather at half the applied frequency which is 0.175 Hz. This phenomenon is seen for a lot of vessels that have an intrinsic contraction frequency less than 0.35 Hz. This suggests that the entrainment seen in the vessels may actually be an effect of shear inhibition when the WSS rises above the critical shear stress. Since the frequency of oscillation of the WSS is more than the vessel’s intrinsic contraction frequency, and since the entrainment is hypothesized to happen as a result of lowering of the intrinsic contraction frequency, it gets entrained at a lower frequency, which is seen to be half the applied frequency. These spectrograms hence show an entrainment of the contraction that is dependent on the applied frequency as well as the intrinsic contraction frequency. Furthermore, the power is higher when the applied pressure gradient is higher, indicating that the entrainment is higher at higher shear stresses.

**Figure 4:**
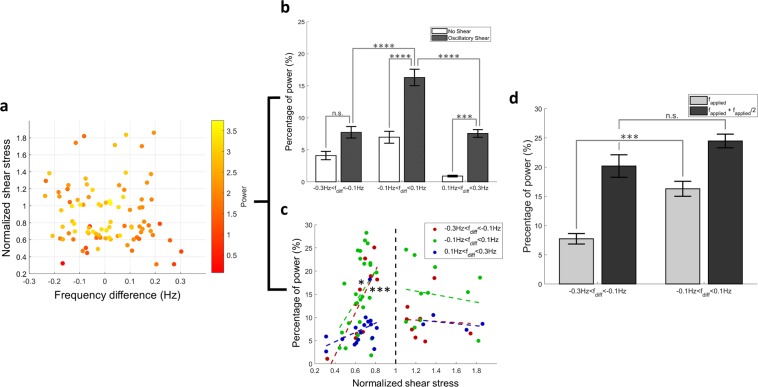
The entrainment of the vessel depends on both the frequency and magnitude of the imposed OWSS. (**a**) The percentage of power representing the entrainment is shown w.r.t. both the frequency difference and the shear stress difference (total N = 94, distributed between the three frequency and three pressure difference conditions). (**b**) The percentage of power is shown at different ranges of frequency under no shear and oscillatory shear conditions, along with the standard error. (**c**) The entrainment is shown w.r.t. to the normalized shear stress (applied shear stress normalized to the critical shear stress for the vessel). The dashed vertical line signifies the threshold at which the applied shear stress is equal to the critical shear stress. The percentage of power is significantly correlated to the normalized shear stress (maximum WSS under imposed oscillatory conditions normalized to the critical WSS for the vessel) when the applied shear stress is below the critical shear stress, for similar and high applied frequencies. (**d**) The percentage of power at the applied frequency is compared to the sum of the percentage of power at the applied and half the applied frequency. When the sum of the percentage of power at both the applied frequency and half the applied frequencies are considered, there is no significant difference between the high and medium applied frequencies. This suggests that more power is present at half the applied frequency components when the externally applied frequency is more than the intrinsic frequency of contraction of the vessel.

The entrainment of thoracic ducts to the applied OWSS was investigated w.r.t. the difference between the intrinsic frequency of contraction and the applied frequency, as well as the applied shear stress normalized to the critical shear stress for the vessel (Fig. 4a). The effect of the frequency difference (calculated as the intrinsic frequency minus the applied frequency) on the entrainment of the vessel to the oscillatory flow was investigated by dividing the dataset into 3 windows corresponding to applied frequencies that were higher than (−0.3 Hz to −0.1 Hz), similar to (−0.1 Hz to 0.1 Hz), and lower than (0.1 Hz to 0.3 Hz) the intrinsic frequency as seen in Fig. 4b. An unbalanced two-way ANOVA test was performed with an alpha level of 0.05 to obtain the statistical significance of the entrainment w.r.t. the frequency difference and the flow conditions (vessels exposed to no shear or OWSS). The entrainment of the vessel to the OWSS is evident from the significantly increased percentage of power in the applied frequency between no shear and oscillatory shear conditions (p = 0.0002, 2.07e-08 and 0.0028 for the three frequency groups). The entrainment was found to be maximum when there is minimal difference between the intrinsic frequency of contraction and the applied frequency. The percentage of power at low applied frequencies was found to be significantly lower than when the applied frequencies were similar to the intrinsic contraction frequency (p = 3.77e-08), demonstrating that the entrainment is hampered at low applied frequencies. To investigate the effect of the magnitude of the applied shear stress on the entrainment, the maximum applied shear stress was normalized w.r.t. the critical shear stress for each vessel so as to incorporate the unique mechanosensitivity of each vessel into the analysis. The entrainment was found to be significantly correlated to the shear stress applied to the vessel (using Fisher’s r-to-z transformation) in the similar and high frequency bands (r-squared values of 0.1643 and 0.6141 with p = 0.0399 and 0.0073 respectively) when the maximum applied shear stress was less than the critical shear stress as seen in Fig. 4d. The entrainment was not significantly correlated to the shear stress for vessels where the maximum applied shear stress was above the critical shear stress, suggesting that there is no added benefit to entrainment from a higher WSS when the WSS is already above the critical shear of the vessel. The possible involvement of shear inhibition as a mechanism for lymphatic entrainment is also supported by Fig. 4d. The significant difference in the entrainment between the applied waveforms with higher and similar frequency to the intrinsic frequency is lost when the power at both the applied frequency and half the applied frequency are considered. This suggests a higher concentration of power at half the applied frequency when the external flow waveform has a higher frequency than the intrinsic frequency and demonstrates that the vessels are contracting at half the applied frequency when the externally applied frequency exceeds the intrinsic frequency.

To determine if vessels other than thoracic ducts might exhibit entrainment properties, preliminary studies with OWSS were performed on two mesenteric lymphatic vessels to test their entrainment. Mesenteric vessels have been shown in the literature to be less sensitive to the same pressure gradient as compared to thoracic ducts. The mesenteric vessels were subjected to oscillatory pressure gradients with an amplitude of 4 cmH_2_O and frequencies of 0.125 Hz and 0.0625 Hz. The diameter and pressure tracings, the CWT of the diameter tracings, and the tracing of the power at the applied and intrinsic contraction frequencies are shown in Supplementary Fig. S1. From the diameter tracings it can be seen that the vessel contraction frequency changes with the application of the oscillatory pressure gradient, and the change is dependent on the applied frequency. This change is reflected by a decrease in the power at the intrinsic frequency and an increase in the power at the applied frequency. However this entrainment to the applied oscillatory pressure gradient is much weaker than that observed in the thoracic duct. The lower entrainment could be attributed to the lower shear sensitivity seen in mesenteric vessels, as compared to thoracic ducts (Fig. S2).

**Figure 5:**
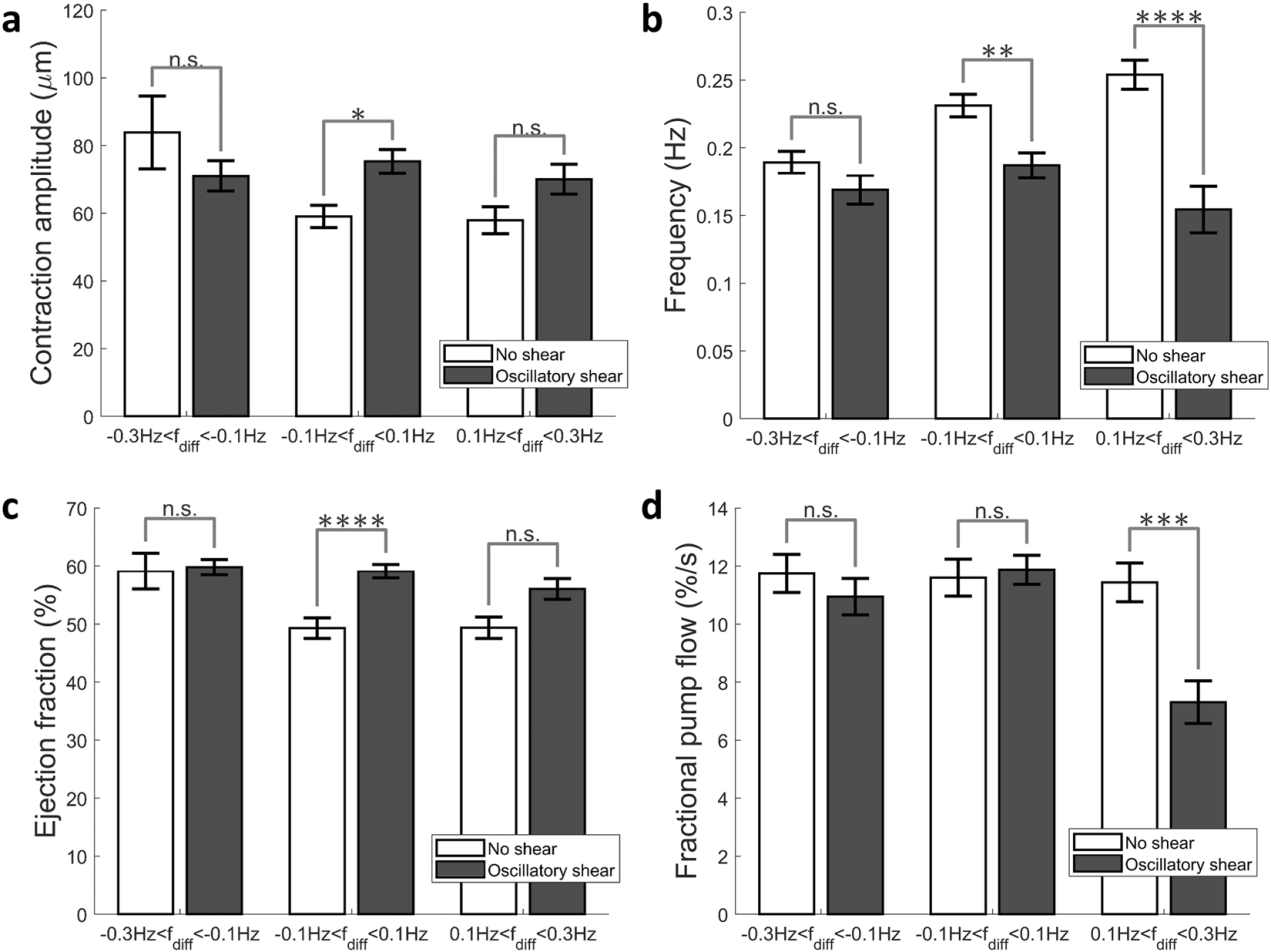
Four parameters of contractility; contraction amplitude, contraction frequency, ejection fraction and fractional pump flow are shown at low, similar and high applied frequency conditions, for both no shear and oscillatory shear cases (N = 94). Ejection fraction and fractional pump flow are lymphatic pumping metrics that represent the percentage of lymph pumped per contraction, and the percentage of lymph pumped per second respectively. (**a**) The contraction amplitude is significantly higher (p = 0.046) for the similar applied frequencies and are not significantly different at other frequencies. (**b**) The contraction frequency is significantly lower for similar (p = 0.0056) and lower (p = 8.86e-08) applied frequencies. (**c**) The ejection is significantly higher (p = 3.84e-05) at similar applied frequencies. (**d**) The fractional pump flow is significantly lower when low-frequency OWSS are applied (p = 2.8e-04).

Four parameters of lymphatic contractility; the contraction amplitude, the contraction frequency, the ejection fraction which represents the percentage of lymph that is pumped by the lymphangion per contraction, and the fractional pump flow which represents the percentage of lymph that is pumped by the lymphangion per second; were investigated as a function of the frequency of the OWSS applied to the vessel and the flow condition, as seen in Fig. 5. An unbalanced two-way ANOVA test was performed with an alpha level of 0.05 to test for the statistical significance of the entrainment w.r.t. the frequency difference and flow conditions. The contraction amplitude (Fig. 5a) was significantly higher in response to the applied oscillatory flow when the applied frequency was similar to the intrinsic contraction frequency (p = 0.046). The contraction frequency (Fig. 5b) was found to be significantly lowered in response to the applied flow for the similar (p = 0.0056) and low (p = 8.86e-08) applied frequencies, with a greater decrease in case of the low applied frequencies. The ejection fraction (Fig. 5c) was found to significantly increase when the applied frequency was close to the intrinsic frequency (p = 3.84e-05). The fractional pump flow (Fig. 5d), representing the fluid pumped as a result of lymphatic contractility (percentage of the fluid volume per second) was found to be significantly lower when the applied frequency was less than the intrinsic frequency (p = 2.84e-04). The increase in the ejection fraction for similar applied frequencies is possibly a consequence of the increased contraction amplitude at those frequencies (Fig. 5a). Since the ejection fraction does not change significantly for the low applied frequency, the lowering of the fractional pump flow at low applied frequencies is possibly a consequence of the contraction frequency of the vessel getting lowered as a result of the imposed OWSS (Fig. 5b).

## Discussion

The lymphatic vessels, owing to their intrinsic contractility, are exposed to a unique mechanical microenvironment that sets them apart from the blood vasculature. Unlike blood vessels, the lymphatic vessels undergo both slow, long-term tonic contractions as well as fast, short-term phasic contractions. This unique contractility subjects the lymphangions to oscillatory transmural pressures and wall shear stresses due to the contractility patterns of upstream and downstream lymphangions. During a typical contraction cycle the lymphangion can contract up to 39% of its diameter and while the average WSS over the contraction cycle is on the order of 0.6 dyn/cm^2^, the peak shear stress is around 8 dyn/cm^2^ as found in rat mesenteric lymphatics^35^. When exposed to edemagenic conditions^36,37^, or in response to elevated loads due to high-fat meals^38^, the flow rate and thus maximal shear experienced by the vessel can increase 10-fold to about 5 to 40 dynes/cm^2^ ^36,37^. In comparison, the blood vasculature of similar dimensions as the lymphatics, such as small arteries in rats typically show average wall shear stress in the order of 15 dynes/cm^2^ ^39^. Smaller arteries and veins can show even higher average shear stress, going up to 70 dynes/cm^2^ ^40–42^. Hence the lymphatic vessels are subjected to a mechanical microenvironment characterized by a low and OWSS, albeit with maximal transient WSS values that can approach that of the blood vasculature.

When exposed to an imposed  pressure gradient *ex-vivo* (in which the imposed WSS was previously unknown), lymphatic vessels are known to inhibit their contraction^16,19^. This shear-dependent inhibition of lymphatic contractility is thought to be an important mechanism in their transition from a pump to a conduit for lymph, depending on the external forces, in order to optimize lymph flow^32^. Shear mediated inhibition of contractility of the lymphangions, similar to the flow-mediated dilation seen in blood vasculature, is endothelium-dependent and has been reported to be dependent on the region chosen for study^33^. *In vitro* studies have also shown the involvement of the lymphatic endothelium in this flow-mediated response^43,44^. However, the actual WSS magnitudes at which this contraction inhibition occurs has been limited to one report on a small set of vessels^13^. As exemplified by Fig. 2, the frequency of contraction of the vessel is found to be inversely related to the shear stress in the vessel. Under sufficiently high shear stresses, the vessel might stop contracting altogether. A critical shear stress can, therefore, be defined as an indicator of a threshold for the pump-conduit transitions for the lymphatic vessels. Further, this critical shear stress is inversely related to the diameter of the vessel, indicating that larger vessels are more sensitive to the wall shear stress. This alludes to an adaptation of the lymphangions to their own unique microenvironment in order to optimize their lymph transport capabilities.

Studies have shown that mesenteric vessels respond less to the same pressure gradient as compared to thoracic ducts^11^. The inhibition of the contraction frequency is not as pronounced for the mesenteric vessels for the same pressure gradient as it is for the thoracic ducts. However these studies did not account for the shear stress on the vessel lumen, which is dependent on the flow rate through the vessel. The flow rate through the vessel is not only dependent on the pressure gradient across the vessel chamber, but the overall resistance of the vessel chamber system. The total resistance of the vessel chamber depends on the vessel diameter, the resistance of the pipette used for the vesel cannulation which is determined by its shape, and the diameter difference between the pipette orifice and the vessel diameter. To determine if the apparently lower sensitivity of the mesenteric vessels is indeed a property of the vessel and not due to the aforementioned discrepancy between the pressure difference and the wall shear stress, a comparison was done between the average shear stress experienced by a thoracic duct and a mesenteric vessel for the same pressure difference. It was found that the shear stress experienced by the mesenteric vessels for the same pressure gradient was in the same order of magnitude as the thoracic duct, and in some cases even higher (Fig. S2). Also, it was seen that the mesenteric vessel contractions were never completely inhibited, and the reduction in contraction frequency at the same shear stress was also less in comparison to the thoracic ducts. The lower entrainment of mesenteric vessels in response to an OWSS (Fig. S1) is also consistent with the lower sensitivity of mesenteric vessels to wall shear stress. Like the thoracic duct, the lower shear sensitivity of mesenteric vessel can translate to a lower percentage of power in the applied frequency. The regional variability in the shear sensitivity of lymphatic vessels and its effect on entrainment of lymphatic vessels to OWSS needs to be investigated further in future work.

The synchronized contraction of the lymphangions in order to pump lymph has long been observed *in vivo* and have been suggested as being important for the optimal transport of lymph depending on the mechanical load that the vessels are being subjected to^45,46^. The lymphatic vessels seem to have developed so as to utilize the propagation of depolarization waves along the lymphatic muscle layer for their contraction, facilitated by the electrical decoupling between the lymphatic endothelial and muscle cell layers^47^. Further, the role of endothelium-derived relaxation factors like nitric oxide and histamine in the flow-mediated dilation have been investigated^19,28,29,31^, and their presence was found to be correlated to the shear stress applied on the vessels. Computational models have also shown that the presence of this mechanosensitivity would facilitate the coordinated pumping of lymphangions^48,49^. Our previous work showed that the application of OWSS can cause entrainment in the contraction of lymphangions, and the entrainment is lost if the shear stress is sufficiently low^13^. Hence the existing literature suggests that the entrainment of contraction between lymphangions is affected at least in part by the shear sensitivity of the lymphangions, thus implicating flow-mediated dilation as a causative factor in the coordinated contraction of a chain of lymphangions.

The idea that entrainment is caused by flow-mediated dilation is supported by the result as represented in Fig. 4 where the percentage of power in the applied frequency, representing the degree of entrainment, was found to be significantly higher when the applied frequency is close to the intrinsic frequency of contraction. This indicates the presence of an optimum frequency of contraction that the lymphatic vessels have developed, possibly as a result of their microenvironment. If the entrainment is a result of inhibition of contraction by flow, it is not possible for the vessel to contract with a frequency higher than its intrinsic contraction frequency. This is supported from our observation that when a frequency of 0.35 Hz is applied to a vessel having an average intrinsic contraction frequency of about 0.3 Hz or lower, the vessel is unable to contract at the imposed frequency, and thus ends up contracting at half the applied frequency, effectively inhibiting its contraction at every other applied OWSS peak. Further supporting a mechanism involving flow-mediated dilation is the observation that the extent of entrainment of the vessel to the imposed flow is highly correlated with the ratio of the maximum imposed wall shear stress and the intrinsic critical shear. The higher the magnitude of the peak shear, the stronger the synchronization. Interestingly, this correlation is lost when comparing vessels in which the peak imposed WSS was above the measured critical shear, suggesting that elevating the WSS stress above this critical shear value has no benefit to further increasing the entrainment.

The response of the lymphangions to an OWSS is hypothesized to be dependent on the lymphatic endothelium. The role of the endothelium in the shear induced entrainment was established in a previous study^13^ where denuded thoracic ducts were found to have an altered response to OWSS. In these experiments the vessel contraction amplitude was hampered significantly as a result of the denudation as can be seen from the diameter tracings in Supplementary Fig. S3. A CWT analysis revealed that the power at the applied frequency was lowered when the vessel was denuded. The power at the applied frequency in the denuded vessels was in the same range as the power in the applied frequency for a vessel with a calcium free media that inhibits all spontaneous contraction, suggesting that any motion at the frequency of the flow waveform is not due to vessel contraction but passive fluctuations in the diameter due to the small transmural pressure fluctuations in the system. However in the absence of an imposed waveform, the denuded vessel still exhibits strong intrinsic contractions, albeit at a reduced frequency. The absence of strong entrained contractions in the denuded lymphatic vessels under flow, even when intrinsic contractions are present under no flow conditions, suggest that the endothelium is required for entrained contraction of the lymphatic vessels to an OWSS.

The shear dependent vasodilation of blood vessels is well documented^50–52^ and similar phenomena have been observed in lymphatic vessels in the form of shear induced inhibition^32,34,53^. The role of the endothelium has been established in the shear induced inhibition, in the form of the involvement of endothelium derived relaxation factors like nitric oxide (NO) and histamine^29,54^. However, NO and histamine may not be the only molecules involved in the shear dependent response of lymphangions. Other molecules such as reactive oxygen species (ROS) have been shown to be implicated in the shear induced mechanotransduction through the endothelium for both blood vessels and lymphatic vessels^54–56^. Further it has been shown that NO is not obligatory for the flow mediated dilation in the radial artery^57^, which alludes to mechanisms other than NO that may be involved in the endothelium-dependent shear induced mechanotransduction. Further studies need to be done in order to delineate the mechanisms for shear induced entrainment, which will be pursued in future work.

The potential physiologic benefit of these unique features of vessel entrainment to flow is perhaps best illustrated in Fig. 5. In chains of lymphangions, one would expect the frequency of the flow waveform resulting from ejection of fluid from the upstream vessel and the frequency of the contractility of the adjacent vessel to be similar, due to the propagation of action potentials along lymphatic muscle cells adjacent to one another, even across the lymphatic valves^58^. Similar contraction frequency between adjacent lymphatics has also been observed *in vivo*^46^ and it has previously been suggested with experimental data that flow-mediated dilation could assist in the coordination of contraction^28^. Thus, the ability to dynamically respond to flow, and the fact that the kinetics of this response is optimized to occur on time scales similar to the vessel’s intrinsic contraction frequency, might provide a mechanism to enhance ejection fraction and optimize lymphatic pumping even in the presence of lymphatic contractile coordination via electrical coupling of lymphatic muscle cells. This is supported by Fig. 5a which shows that there is a significant increase in the ejection fraction when the externally applied flow is oscillating at a frequency close to the intrinsic frequency of contraction of the vessel. Similarly Fig. 5b shows a significant reduction in the fractional pump flow when the applied flow frequency is much lower than the intrinsic frequency of the lymphangion, due to the externally applied flow lowering the contractile frequency below its intrinsic value. Computational modeling that incorporates these dynamic responses, with values from actual experimental data such as that reported here, could shed light on the benefit of this flow-mediated entrainment, possibly by preserving the energy expenditure by lymphatic muscle cells and enhancing overall lymph transport.

The literature has also shown that the mechanosensitivity of lymphangions to shear stress is hampered in pathological states that have been related to impaired lymphatic transport, such as those that might occur in the case of metabolic syndrome^53,59^ and as a result of aging^12,60^. The entrainment of lymphangions to shear stress may also be hampered during cases of lymphatic endothelial dysfunction that affects flow-mediated dilation, thus leading to deficient pumping in the lymphatics or the unnecessary expenditure of energy by lymphatic muscle cells. This could provide a mechanism for reduced lymphatic pump flow during conditions of lymphatic dysfunction, possibly leading to impaired tissues-fluid homeostasis and other complications that may arise due to a compromised lymphatic system. Thus the physiologic consequences of impaired flow-mediated dilation to lymphatic pump performance in the context of disease is an important area of future study.

## Conclusions

The lymphatic vessels are highly attuned to their local mechanical microenvironment as is reflected by the dependence of the shear sensitivity of the lymphatics on the diameter of the vessel. The shear-dependent inhibition leads to an entrained contraction of the lymphangions to an oscillatory stimulus, thus pointing to a physical mechanism by which the coordination of pumping between lymphangions might occur, leading to optimized lymph flow. The molecular mechanisms regulating this entrainment are not completely understood and future work should investigate the role of endothelial-derived relaxation factors on this entrainment. Computational models can be improved with the information about the dependence of shear sensitivity on the diameter of the vessel, which can then be used to investigate how the coordination between lymphangions can affect the lymphatic pump function and how this might be compromised in disease.

## Methods

### Experimental Setup

**Figure 6:**
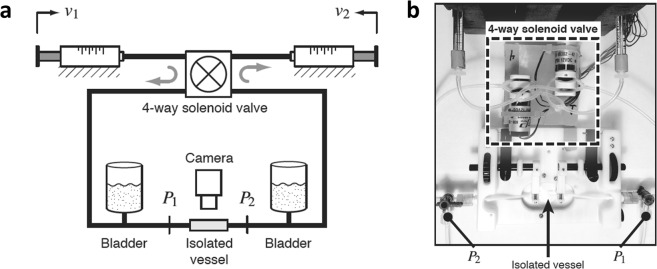
The vessel perfusion system is shown, highlighting the major components. (**a**) The basic control scheme of the perfusion device is shown in the cartoon^61^. The syringes ensure flow through the system and help maintain the inlet and outlet pressures. The solenoid valves ensure unidirectional flow through the chamber, from the inlet to the outlet. Pressure transducers P_1_ and P_2_ record the pressure at the inlet and outlet of the vessel chamber. A feedback loop, using the pressure readings, ensures that the inlet and outlet pressures are maintained at the desired value. (**b**) The top view shows the tubing connections from the perfusion system to the vessel chamber, the location of the isolated vessel in the chamber and the inlet and outlet ports of the vessel chamber.

The experiments were performed on thoracic ducts isolated from male Sprague-Dawley rats that weigh between 280–300 gm. All procedures performed on the rats were reviewed by the Institutional Animal Care and Use Committee (IACUC) at Georgia Institute of Technology. The proposed procedures were found to comply with the Institute’s Policy for Humane Care and Use of Laboratory Animals, and were approved by the IACUC at Georgia Tech (protocol number A14069). The thoracic duct was chosen since it has been observed that they show more sensitivity to shear stress variations than mesenteric lymphatic vessels^34^. Vessel segments devoid of lymphatic valves were chosen for the studies to avoid nonlinearities that may impact the performance of the isolated vessel perfusion system. The isolated vessels were cannulated in a vessel chamber and immersed in and perfused with a physiological salt solution (PSS). The experimental setup consists of a commercially available vessel chamber from Living Systems Instrumentation, connected to a custom perfusion system that allows the independent control of transmural pressure (P_avg_) and pressure gradient (ΔP) along the vessel chamber through explicit model predictive control^61^ as shown in Fig. 6. The whole setup was mounted on an inverted microscope, which was used to capture bright-field images of the vessel as it was exposed to the various flow conditions. Two micropipette tips mounted on the vessel chamber were used to cannulate the isolated thoracic duct. The size of the thoracic ducts ranged between 500 μm to 900 μm. Keeping this in mind, pipette tips that are approximately 450 μm in diameter were chosen. The vessel chamber was heated using electric heating pads and the temperature was maintained around 38 °C using a thermocouple and a temperature controller. To ensure that the composition of the media did not change during the course of the experiments, fresh media was recirculated in the chamber using a peristaltic pump, running at a flow rate of 0.3 mL/min.

The mechanical conditions inside the vessel were controlled by the perfusion system that utilizes linear motors, gas-tight syringes and solenoid valves to control the pressures at the inlet and outlets of the vessel chamber, P1 and P2 respectively^61^. Analog pressure transducers were used to detect these pressures and control the device. The transmural pressure was calculated as the average of the inlet and outlet pressures, (P1 + P2)/2. The pipette tips were resistance matched and the length of the tubes connecting each pipette to the external tubing was matched to ensure that the pressure drop in both pipettes are the same. The resistance matching ensures that there is no flow through the vessel when the pressure at the inlet and outlet of the vessel chamber are the same, or in other words, there is no offset in the relationship between the pressure gradient across the vessel and (P1-P2).

From the average pressure and pressure difference that are applied to the vessel, it can be seen that in certain instances, the pressure at one of the ends of the vessel chamber can drop to below 0 cmH_2_O. However, it is to be noted that the pressures recorded by the transducers were at either end of the vessel chamber, which is not the same as the pressure at the end of the cannulated vessel itself. The pressure drop across the vessel can be calculated from a knowledge of the diameter and length of the vessel, and the flow rate through the vessel, assuming a Poisuielle approximation for low Reynold’s number flow. Assuming a vessel with a diameter of 500 µm (considering a thoracic duct) and a peak flow rate of 9 µL/s through the lumen (representative flow rate calculated from experimental recordings of the syringe positions), the wall shear stress produced is about 5 dynes/cm^2^, which is in the physiologic range for the thoracic ducts. The pressure drop across a vessel segment with a length of 5 mm (representative length of vessels that were used for the experiments) is about 0.2 cmH_2_O for the aforementioned mechanical conditions, which is an order of magnitude lower than the total pressure drop across the system of about 4–8 cmH_2_O. Hence the pressure at either end of the vessel never reaches 0 cmH_2_O even during peak flow, thus eliminating the concern of the vessel collapsing during any of the experiments. Visually, the vessels were never seen to collapse during the experiment as well.

### Protocols

Each vessel was first stepped through average transmural pressures (P_avg_) of 1, 3, 5 and 7 cmH_2_O under zero pressure gradient (ΔP) over 12 minutes to pre-condition them. This was done to ensure that the vessel isolation and cannulation process did not damage the vessel and that reference contraction frequency for the vessel can be determined, which is useful in the subsequent steps. After pre-conditioning, the vessel was subjected to 5 minute and 15-minute ΔP ramps from 0–9 cmH_2_O at a P_avg_ of 3 cmH_2_O. The vessels were then taken through a series of oscillatory flow waveforms with different frequencies and amplitudes. Each step consists of 5 minutes of zero ΔP, and then 5 minutes of oscillatory ΔP, at a P_avg_ of 3 cmH_2_O. This P_avg_ was chosen as 3 cmH_2_O since the contractile function of the thoracic duct (pumping frequency and stroke volume) was found to be optimum at this pressure. At no point was the vessel subjected to a retrograde flow. The frequencies of the applied flow waveforms were 0.075 Hz, 0.2 Hz, and 0.35 Hz. These frequency values were chosen to correspond to frequencies much less than, similar to, and much greater than the intrinsic contraction frequency of about 0.3 Hz so that an even distribution of data as a function of frequency is obtained during the data analysis. The amplitudes of the ΔP were chosen to be 4, 6 and 8 cmH_2_O in order to correspond to “low”, “medium” and “high” shear stress as compared to the critical shear stress at which inhibition occurs (typically between 3–7 cmH_2_O for the rat thoracic duct). This choice of the ΔP also ensured an even distribution of data as a function of shear stress during the data analysis.

### Data Analysis

**Figure 7:**
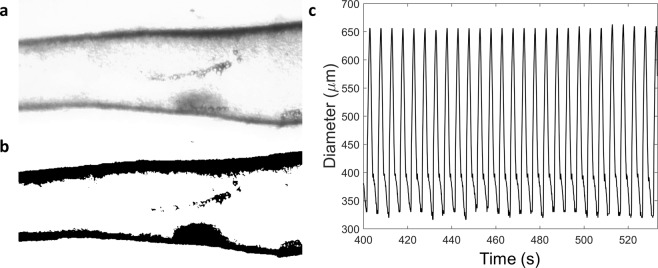
The data analysis used to obtain the diameter tracings from the brightfield images is shown. (**a**) Brightfield images of the vessels were acquired using an inverted microscope. (**b**) Binary images of the vessel, isolated from the background were obtained by thresholding the brightfield images. The binary images were used to detect the diameter of the vessel at different locations along the vessel. (**v**) Going through all the frames in the video, the diameter was obtained as a function of time for all the locations along the vessel.

The data was acquired in the form of brightfield videos of the vessel acquired at 4x magnification. The videos were then processed frame by frame by a window-based thresholding algorithm that helped distinguish the vessel from the background as shown in Fig. 7a,b. Processing was also done at this step to remove any noise due to particles floating in the chamber. The diameter at each location along the vessel was then calculated by detecting the upper and lower boundaries of the vessel in the thresholded image. The diameters were detected over time to form the diameter tracings (Fig. 7b) that were then subsequently analyzed at each location along the vessel. Once the diameter data was obtained as a function of time and location, the tracings were filtered using an averaging low-pass filter to remove any noise due to the resolution of the image.

The diameter tracings were then analyzed with Continuous Wavelet Transform (CWT). CWT is a frequency based analysis technique that allows for the decomposition of a signal into its constituent components at different frequencies and times. The most commonly used tool in the analysis of frequency domain information is the Fourier Transform (FT), but it shows the magnitude of the frequency content of the data over a time window. When the spectral information is needed as a function of time, a modification to this method can be used which is called the “Short Time Fourier Transform” (STFT). In this technique, the spectral information is obtained over user-defined time windows within the data, where the windows overlap with each other by an amount defined by the user. Hence, it is apparent that in this method, the temporal resolution is limited by the amount of overlap, and the user-defined nature of the time window can change the amount of information captured. For example, a smaller time window can be used when the data is rich in higher frequencies and a larger window should be used when the frequency in the data is more concentrated in the low frequencies, so as not to miss out on any information. These problems can be avoided using Continuous Wavelet Transforms as it accounts for the varying time and frequency resolution without any user intervention, thus reducing human interference to the analysis. It provides higher temporal and frequency resolution, thus enabling the study of the spectrum of the signal with higher fidelity.

The coefficient of a CWT is a representation of the magnitude of a component at that particular frequency and time. The higher the magnitude of the CWT coefficient at a specific frequency (referred to as power) and time, the greater the magnitude of that frequency component at that particular time. Hence the power at the applied frequency is also dependent on the contraction amplitude of the vessel at a particular time. However, the entrainment of the vessel to the OWSS should be a non-dimensional quantity that is independent of the contraction amplitude. The entrainment of the vessel is a measure of the relative importance of the frequency component at the applied OWSS over other frequency components that may be present in the diameter tracings. Hence the percentage of power at the applied frequency at a particular time is defined as a non-dimensional measure of the entrainment, since it is independent of the diameter of the vessel and is representative of the relative magnitude of the applied frequency component compared to the other frequency components.

Time domain techniques to analyze entrainment, namely looking at only the average contraction frequency, only provides binary information regarding entrainment without providing any information about how the OWSS parameters affect the degree of entrainment. Time domain techniques also do not provide any information about the relative presence of a particular frequency component in the overall signal, which is crucial in order to define a non-dimensional measure of entrainment as mentioned before. Hence CWT provides an essential tool to study the amount of entrainment of a lymphangion to a spectrum of OWSS waveforms with different frequencies and amplitudes.

### Calculation of Wall Shear Stress

The pressure difference across the vessel chamber is not a reliable estimate of the wall shear stress on the lumen of the vessel. There are minor losses associated with the sudden expansion of the pipette tip into a much larger vessel lumen. The pipette tip diameter is fixed, but the vessel diameter is variable, and hence the effective pressure drop across the vessel becomes a function of the vessel diameter. Hence the shear data for these experiments were obtained from the information of the location of the syringes as a function of time. Due to noise in the location data of the syringe positions, the locations of the syringes were tracked in 3-second intervals to obtain an average syringe velocity over that time interval. After this time averaged syringe velocity (V_avg_) was obtained, the flow rate, Q, was calculated from the knowledge of the inner radius, r of the syringe as

$$Q=\pi {r}^{2}{V}_{avg}$$

From the diameter tracings of the vessel, an average diastolic diameter of the vessel, D_diast_ was calculated. Using this diameter value, the representative wall shear stress was calculated as

$${\tau }_{wall}=\frac{32\mu Q}{\pi {D}_{diast}^{3}}$$

Where μ is the dynamic viscosity of water at 38 °C which is around 6.78 mdyn-s/cm^2^. Given the low Reynolds number (i.e. Re ≪ 1) and low Womersley number (Wo < 0.1) for these flows^62^, and the fact that relatively straight vessel segments without valves are chosen, the Poisuielle flow approximation could be used to get an appropriate estimate of the applied WSS.

**Figure 8:**
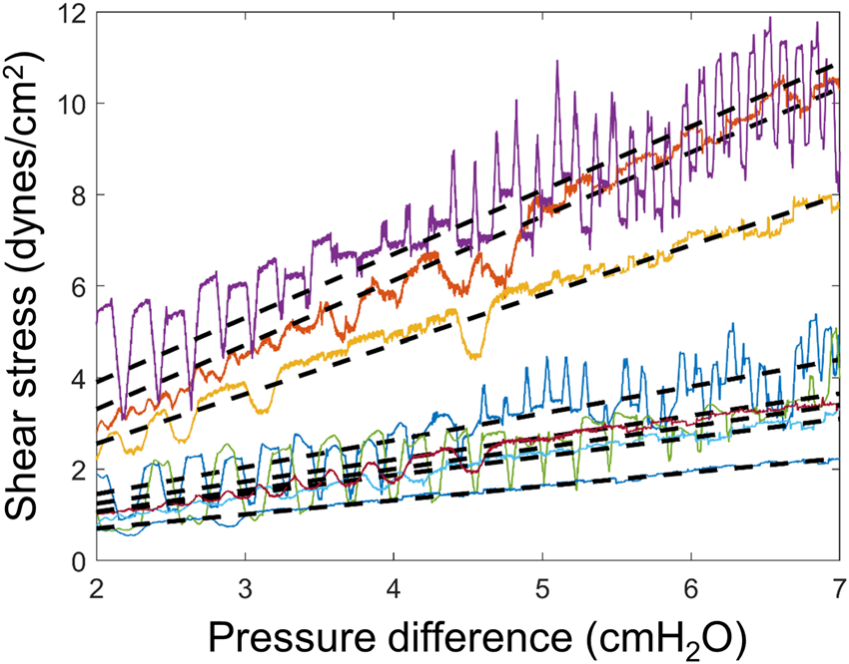
For the ramp shear stress experiments, the wall shear stress is plotted as a function of the pressure difference. The 8 colored plots represent 8 different vessels on which the ramp protocol was applied. The dashed lines represent linear fits to the shear stress vs pressure difference plots. The linear fit between the shear stress and the pressure difference across the system was used to convert between the pressure difference and shear stress for the oscillatory flow experiments.

Once the shear stress data was obtained for ramp experiments, it was used to convert the ΔP to WSS to be used in the conditions where an OWSS was applied. This conversion is required since the syringe position is averaged over 3 seconds to be reliable, a time window that is too long to capture the shear stress variations in high-frequency oscillatory flow conditions. This estimation was done by obtaining the ramp shear stress as a function of ΔP and then plotting straight lines to them, as shown in Fig. 8. The pressure to WSS relationship was obtained for each vessel individually. The slope and offset of the line fits were used to calculate the OWSS from the oscillatory ΔP for each vessel. The advantage of calculating the OWSS in this way is that there is no reliance on the ΔP across the system for calculating the WSS, hence eliminating the variability introduced in the signal due to changes in the vessel diameter.

## Supplementary information

Supplementary Figures
