# Supplementary material for: Entrainment of Lymphatic Contraction to Oscillatory Flow
Source: Sci Rep. 2019 Apr 9;9:5840. doi: 10.1038/s41598-019-42142-9 (PMC6456495; doi:10.1038/s41598-019-42142-9)
Supplement: Supplementary file 1 — Supplementary Figures [file 41598_2019_42142_MOESM1_ESM.docx]

**Supplementary Information**

**Entrainment of Lymphatic Contraction to Oscillatory Flow**

Anish Mukherjee^1^, Joshua Hooks^2^, Zhanna Nepiyushchikh^2^, J. Brandon Dixon^2,3^
^1^Department of Electrical and Computer Engineering, ^2^Department of Mechanical Engineering, ^3^Department of Biomedical Engineering, Georgia Institute of Technology, Atlanta, GA 30332

**Supplementary Figure S1**


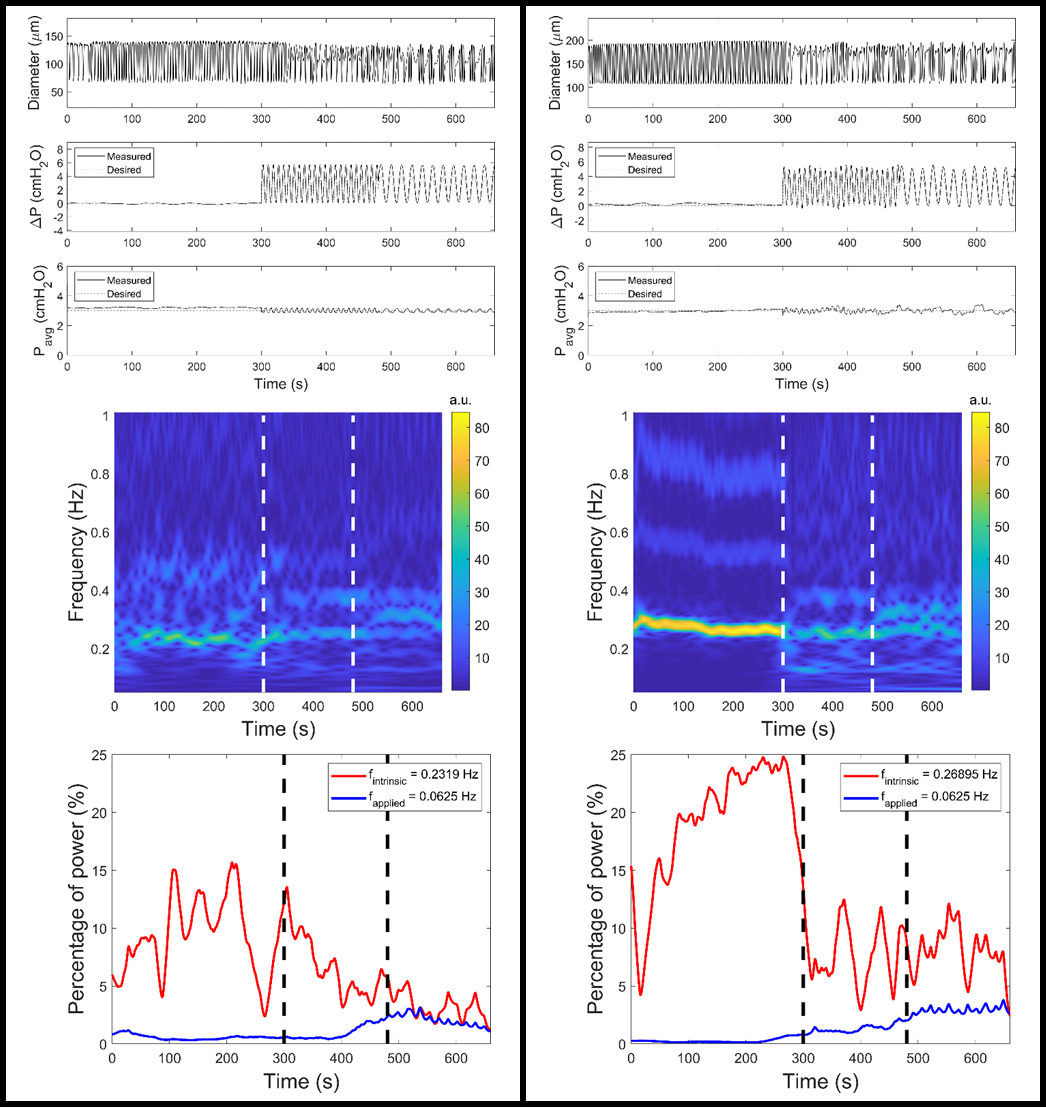


Fig. S1: The response to oscillatory pressure waveforms have been shown for two mesenteric lymphatic vessels. The top two sets of tracings show the diameter tracings along with the pressure gradient and average pressure tracings. The lower two images represent the CWT of the diameter tracing and the plot of the power at one of the applied frequencies vs time, respectively. The CWT and power tracings have been divided by the dashed lines into 3 sections: the first section corresponds to no flow (intrinsic contractility) condition, the second section had an oscillatory pressure waveform with an amplitude of 4 cmH_2_O and frequency of 0.125 Hz applied to the vessel, and the third section had an oscillatory pressure waveform with amplitude of 4 cmH_2_O and frequency of 0.0625 Hz applied to the vessel.

**Supplementary Figure S2**


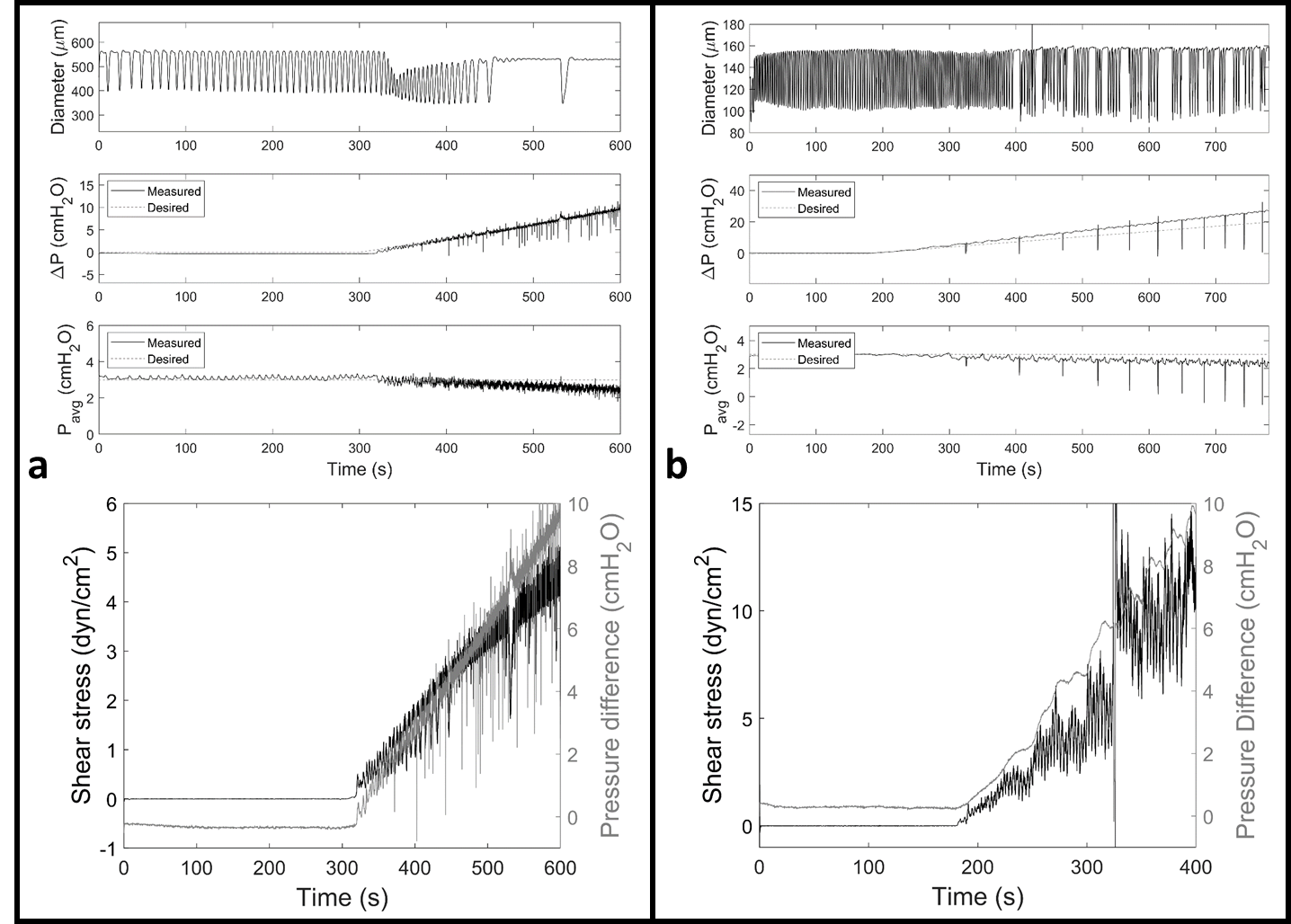


Fig. S2: The response to a ramped pressure gradient, along with the shear stresses have been shown for a) a thoracic duct and b) a mesenteric lymphatic vessel. The top graphs show the diameter tracings along with the pressure gradient and average pressure tracings. The lower two graphs show the wall shear stress on the vessel lumen plotted on top of the pressure gradient applied across the vessel chamber.

**Supplementary Figure S3**


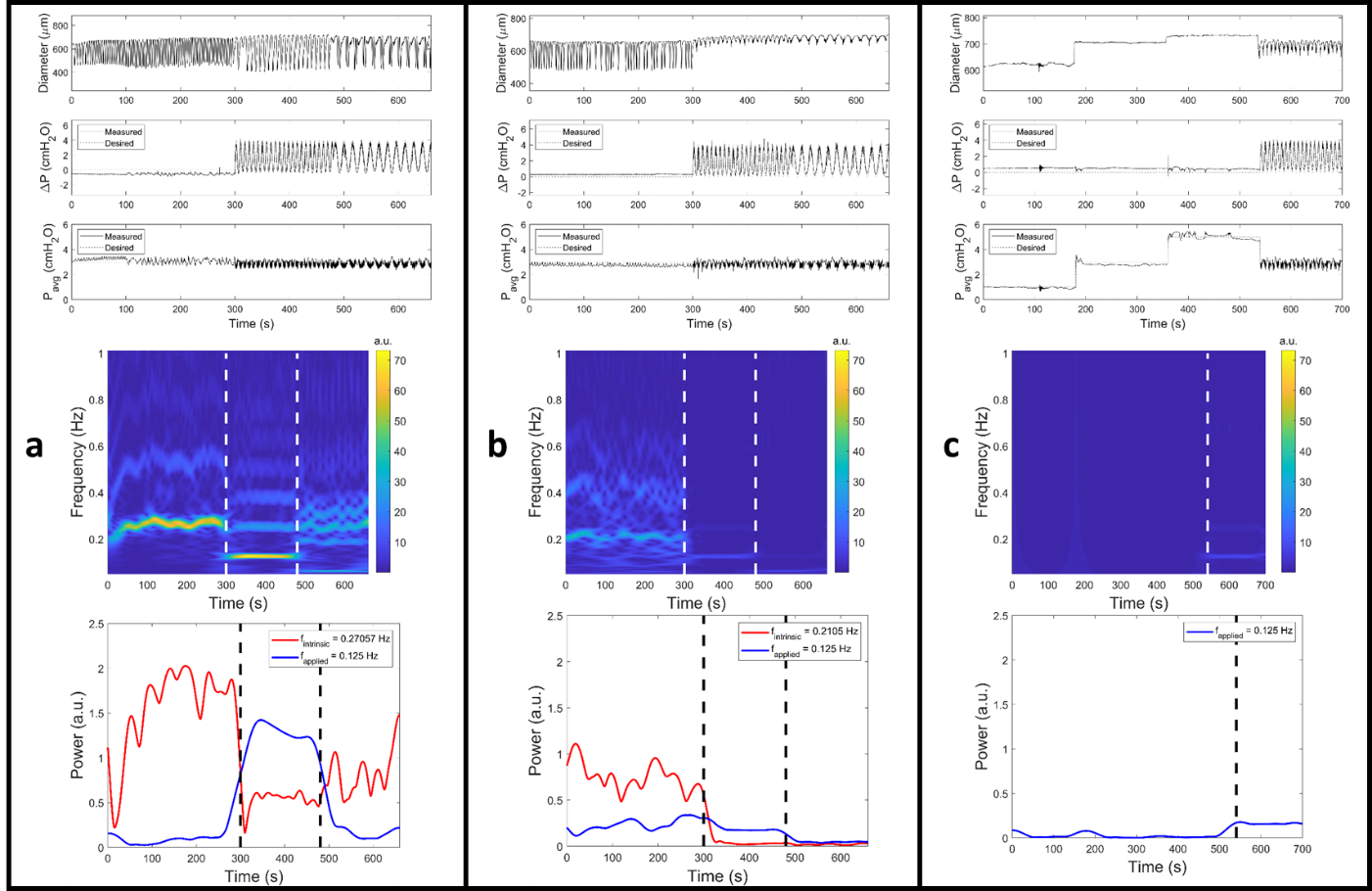


Fig. S3: The diameter and pressure tracings, CWT of diameter tracings, and power in the applied frequency vs time are shown in the top, middle and bottom panels respectively. The intact vessel (a) and denuded vessel (b) are exposed to 3 pressure conditions differentiated by the dashed lines: the first section corresponds to no flow (intrinsic contractility) condition, the second section had an oscillatory pressure waveform with an amplitude of 4 cmH_2_O and frequency of 0.125 Hz applied to the vessel, and the third section had an oscillatory pressure waveform with amplitude of 4 cmH_2_O and frequency of 0.0625 Hz applied to the vessel. The denuded vessel exposed to calcium free media (c) was taken through transmural pressure steps of 1, 3 and 5 cmH_2_O at 0 cmH_2_O pressure gradient (no flow) and subsequently exposed to an oscillatory pressure gradient with an amplitude of 4 cmH_2_O and frequency of 0.125 Hz.
